# Temporal covariance structure of multi-spectral phenotypes and their predictive ability for end-of-season traits in maize

**DOI:** 10.1007/s00122-020-03637-6

**Published:** 2020-07-01

**Authors:** Mahlet T. Anche, Nicholas S. Kaczmar, Nicolas Morales, James W. Clohessy, Daniel C. Ilut, Michael A. Gore, Kelly R. Robbins

**Affiliations:** 1 Plant Breeding and Genetics Section, School of Integrative Plant Science, Cornell University, Ithaca, NY 14853 USA; 2 Present Address: Horticulture Section, School of Integrative Plant Science, Cornell University, Ithaca, NY 14853 USA; 3 Present Address: North Florida Research and Education Center, Plant Pathology Department, University of Florida, Quincy, FL 32351 USA

## Abstract

**Key message:**

Heritable variation in phenotypes extracted from multi-spectral images (MSIs) and strong genetic correlations with end-of-season traits indicates the value of MSIs for crop improvement and modeling of plant growth curve.

**Abstract:**

Vegetation indices (VIs) derived from multi-spectral imaging (MSI) platforms can be used to study properties of crop canopy, providing non-destructive phenotypes that could be used to better understand growth curves throughout the growing season. To investigate the amount of variation present in several VIs and their relationship with important end-of-season traits, genetic and residual (co)variances for VIs, grain yield and moisture were estimated using data collected from maize hybrid trials. The VIs considered were Normalized Difference Vegetation Index (NDVI), Green NDVI, Red Edge NDVI, Soil-Adjusted Vegetation Index, Enhanced Vegetation Index and simple Ratio of Near Infrared to Red (Red) reflectance. Genetic correlations of VIs with grain yield and moisture were used to fit multi-trait models for prediction of end-of-season traits and evaluated using within site/year cross-validation. To explore alternatives to fitting multiple phenotypes from MSI, random regression models with linear splines were fit using data collected in 2016 and 2017. Heritability estimates ranging from (0.10 to 0.82) were observed, indicating that there exists considerable amount of genetic variation in these VIs. Furthermore, strong genetic and residual correlations of the VIs, NDVI and NDRE, with grain yield and moisture were found. Considerable increases in prediction accuracy were observed from the multi-trait model when using NDVI and NDRE as a secondary trait. Finally, random regression with a linear spline function shows potential to be used as an alternative to mixed models to fit VIs from multiple time points.

## Introduction

In the past few decades, advances in genotyping and computational technologies have contributed greatly to the genetic improvement in crops. In plant breeding, however, the rate of genetic gain that can be achieved is hampered by the high cost and time-consuming nature of phenotyping (Reynolds et al. 2019). This is especially true when phenotyping needs to be conducted for a large number of phenotypes in large-scale field trials over multiple geographical locations. Recent advances in multi-spectral imaging (MSI) platforms and image processing techniques have emerged, allowing for the generation of high-dimensional phenotypic data for plant breeders. MSI serves as an efficient, non-destructive approach to monitor the properties of crop canopies over time and space.

Canopy spectral reflectance has been successfully used to measure phenotypes for different crops, such as triticale (Busemeyer et al. 2013), wheat (Crain et al. 2016), cotton (Pauli et al. 2016), soybean (Bai et al. 2016) and maize (Herrmann et al. 2020). Different spectral readings from MSI platforms can then be combined to produce vegetation indices (VIs) that describe the crop over the growing season. Among different VIs, Normalized Difference Vegetation Index (NDVI) is calculated using the near infrared (NIR) and visible red (R) reflectance. Deering (1978) suggested NDVI as a measure of leaf area index (LAI). As reported by Babar et al. (2006), larger NDVI values are associated with greater biomass accumulation and a faster growth rate when measured during the vegetative stage of plant growth. However, recent studies suggest that NDVI is a better estimator of light interception by canopies due to the fact that NDVI values get saturated during the growing season (Hatfield and Prueger 2010). In addition to NDVI, Green NDVI (GNDVI), Red Edge NDVI (NDRE), Soil-Adjusted Vegetation Index (SAVI) (Huete 1988) and Enhanced Vegetation Index (EVI) are VIs that are used to measure LAI.

Phenotypic analysis of NDVI and their association with maize grain yield and biomass has been assessed by Tattaris et al. (2016). Even though no genetic analysis was performed, Tattaris et al. (2016) found a strong phenotypic correlation of UAV-measured NDVI with grain yield and biomass. Studies on wheat also reported that NDVI is genetically correlated with grain yield (Labus et al. 2002; Mason and Singh 2014). Moreover, it was reported that Blue NDVI can be used to predict maize flowering time, yield and kernel dimension (Wu et al. 2019). Hence, if the VIs derived from MSI are found to be heritable and genetically correlated with economically important traits, they can be used to assist in phenotypic or genomic selection.

Another advantage of MSI is that it permits the collection of repeated measurements at multiple time points during the growing season. The ability to monitor the crop throughout the growing season provides potential insight into the interaction of a genotype with the environment, which could improve our understanding of genotype-by-environment (*G* × *E*) interactions observed for end-of-season traits such as grain yield. Rapid changes in (co)variance parameters between adjacent time points and end-of-season traits would be indicative of significant environmental and/or plant developmental events. The use of covariance functions for estimation of changes in covariance would enable the focused examination of *G* × *E* interactions across a wide number of trials. Furthermore, heritable parameters of growth curves could provide breeders with additional information to select for genotypes with optimal growth curves for a given set of target environments. The challenge with these types of analyses is the ability to fit parsimonious models that accurately model continuous changes in covariance parameters across the entire growing season.

Repeatability and multi-trait mixed models have been used to model repeated measurements that are recorded through time (Mrode and Thompson 2005; Speidel et al. 2010). The repeatability model, however, assumes constant variance and covariance among and at multiple time points. In multi-trait mixed models, assumptions about the pattern of (co)variance can be relaxed, but with a large number of time points the number of parameters that must be estimated will not be scalable. Additionally, the high correlation between consecutive records can create computational issues. As an alternative to repeatability and multi-trait mixed models, random regression models have been implemented to model such repeated measurements that are recorded in a continuous scale, such as time or age (Kirkpatrick et al. 1990; Huisman et al. 2002; Boligon et al. 2012; Lopes et al. 2012; Brito et al. 2017). Random regression models commonly use Legendre polynomials to model the variance and covariance of measurements at and among the time points (Meyer 2005). However, it has been noted that such high-order polynomials are problematic at extreme values when data are sparse, resulting in poor estimation of variance and covariance (Misztal 2006). As an alternative to such high-order polynomials, splines, which are piecewise functions consisting of segments that are connected by so-called knots, have gained popularity for analyzing repeated measurements in random regression models when data may be sparse or highly clustered (Robbins et al. 2005; Meyer 2005; Bohmanova et al. 2008).

The objectives of this study were to investigate the amount of variation present in several vegetation indices (VIs) derived from MSI and the changes in (co)variance parameters through time and assess the potential use of these indices as an indicator trait for grain yield and moisture using a multi-trait model. Finally, we aimed to investigate the potential of a random regression model to fit multiple phenotypes from MSI as an alternative to the mixed model.

## Materials and methods

### Agronomic phenotypic data

The phenotypic data were collected from trials at Cornell University’s Musgrave Research Farm as part of the Genomes to Fields (G2F) initiative (http://datacommons.cyverse.org/browse/iplant/home/shared/commons_repo/curated/GenomesToFields_2014_2017_v1).

The trials were planted at two sites in Aurora, New York (NYH2 and NYH3), from 2015 to 2017 (Alkhalifah et al. 2018; McFarland et al. 2020). The field trial was planted as a randomized complete block design with two replicates at each location as two-row plots. In 2015, plot-level phenotypic data were collected for a number of important end-of-season traits for 375 maize hybrids at NYH2 and 367 hybrids at NYH3, with an overlap of 253 hybrids. In 2016 and 2017, phenotypic data were available for 195 and 184 hybrids, respectively, with an overlap of 173 hybrids. However, there were less than 10 hybrids that overlapped between the 2016/2017 and 2015 data. For 2016 and 2017, phenotypic data only from NYH2 sites were used. This is because image data were available only for the NYH2 field site. Due to poor weather conditions, experiments in 2016 could not be machine harvested. For that reason, the end-of-season trait data for this year were not used. However, the MSI data were of sufficient quality and thus were used to investigate the amount of variation present in different VIs and to assess the potential of random regression model as an alternative to mixed models.

For this study, all analyses were nested within sites and year to avoid issues aligning MSI data collected from experiments with different planting and drone flight dates.

### UAV data collection and image processing

For 2015 and 2016, aerial survey flights were conducted by Flyterra (Quebec, CA) (http://flyterra.com/?page_id=388). A Microdrones MD4-1000 unmanned aerial vehicle (UAV) equipped with a Micasense RedEdge 3 multi-spectral camera was used for the aerial surveys. The Micasense RedEdge is a multi-spectral sensor that is comprised of 5 individual 1.2MP 12 bit sensors, each used to detect specific wavelengths (Blue: 460–480 nm, Green: 550–560 nm, Red: 660–670 nm, Red Edge: 710–720 nm, and NIR: 840–860 nm). The UAV flew via GPS-guided autopilot using programmed grid survey patterns across the experimental plots at a flight altitude of 75 m (5 cm/pixel GSD). There were four and five aerial survey flights conducted in 2015 and 2016, respectively.

In 2017, six aerial survey flights were conducted by a certified remote pilot operating under FAA Part 107 guidelines. A DJI Matrice 600 equipped with a DRTK-GPS guidance system and a Micasense RedEdge 3 were used for the aerial surveys. The programmed grid survey patterns were flown using an application called Litchi across experimental plots at a flight altitude of 25 m (2 cm/pixel GSD). Images of the Micasense calibration panel were taken before and after each flight in 2015–2017. Orthomosaics from each aerial survey flight were constructed in Pix4Dmapper (https://www.pix4d.com), which were then used to calculate summary statistics, such as mean, median and variance for individual bands/reflectance for each plot. For 2017, the second time point was removed due to model convergence issues in the univariate analysis.

Using these summary statistics, multiple VIs were calculated for each plot using the following equations:

1 $${\text{NDVI}} = \frac{{\left( {R_{\text{NIR}} - R_{\text{R}} } \right)}}{{\left( {R_{\text{NIR}} + R_{\text{R}} } \right)}}$$

where $R_{\text{NIR}}$ is the near infrared reflectance and $R_{\text{R}}$ is the red reflectance. GNDVI and NDRE were calculated using green and red edge reflectance instead of the red reflectance in Eq. 1, respectively. SAVI and EVI, which correct for the soil background, were calculated following Hatfield and Prueger (2010);

2 $${\text{SAVI}} = \frac{{\left( {R_{\text{NIR}} - R_{\text{R}} } \right)\left( {1 + L} \right)}}{{\left( {R_{\text{NIR}} + R_{\text{R}} + L} \right)}} ,$$

where *L* is the soil background correction factor and *L* = 0.5 was used in this study (Hatfield and Prueger 2010);

3 $${\text{EVI}} = 2.5\left( {R_{\text{NIR}} - R_{\text{R}} } \right)/\left( {R_{\text{NIR}} + 6R_{\text{R}} - 7.5R_{\text{B}} + 1} \right)$$

A simple Ratio was calculated as

4 $${\text{Ratio}} = \frac{{R_{\text{NIR}} }}{{R_{\text{R}} }}$$

Among different summary statistics that were available for the VIs, the mean values for each plot were used for the analysis.

The VIs are calculated as a ratio that falls between 0 and 1, and tended to be negatively skewed. For that reason, log base 10 transformation was done for the VIs at each time point to reduce skeweness and results were compared to analysis on the untransformed data. Given that the transformation had no impact on the mixed model results, the analysis was performed on the untransformed phenotypic values.

Table 1 summarizes the dates, growing degree days (GDD) and estimated growth stages at which MSI phenotypes were collected. Based on planting and silking dates at each site/year, all MSI in 2015 and 2017 at NYH2 was collected at the reproductive stage. In 2015 at NYH3 and in 2016 at NYH2, the first time point captured the late vegetative stages, while the rest of the imaging dates captured the reproductive stages. Hence, the time points at each site/year are classified as late vegetative stage (VT), early (R1–R2), mid (R3–R4)- and late (R5–R6) reproductive stages.

**Table 1:** Planting, silking, multi-spectral Imaging (MSI) dates, growing degree days (GDDs) and growth stages across sites/years

Planting date	2015 NYH2		2015 NYH3			2016 NYH2			2017 NYH2		
	May 07		May 23			May 10			May 18		
Silking date	July 17–August 06		July 26–August 22			July 10–August 14			July 17–August 05		
MSI date	GDD	Growth stage	MSI date	GDD	Growth stage	MSI date	GDD	Growth stage	MSI Date	GDD	Growth stage
07/20	686	R1–R2 R1–R2	07/20	554	VT	07/05	524	VT	08/02	814	R1–R2
08/07	882		08/07	759	R1–R2 R1–R2	07/27	816	R1–R2	08/17	974	R3–R4 R3–R4 R3–R4
08/20	1037	R3–R4	08/20	916		08/19	1129	R3–R4	09/01	1102	
09/10	1272	R5–R6	09/10	1157	R3–R4	08/23	1173	R3–R4	09/06	1135	
						09/21	1501	R5–R6	09/12	1167	R5–R6
									09/24	1179	R5–R6

VT = late vegetative stage, R1–R2 = early, R3–R4 = mid, and R5–R6 = late reproductive stage

### Genotypic data

Genotyping-by-sequencing (GBS) data scoring 600 K single-nucleotide polymorphism (SNP) markers were available for 1557 parental lines (http://datacommons.cyverse.org/browse/iplant/home/shared/commons_repo/curated/GenomesToFields_2014_2017_v1/G2F_Planting_Season_2017_v1). Quality control for markers was performed as follows: SNP markers with > 15% missing data, minor allele frequency < 0.01 and with % heterozygosity > 1 were removed. Missing genotypes were imputed using the population mode. The remaining 122 K SNP markers were used to calculate the additive genomic relationship matrix between the hybrids. Additive genomic relationship matrix for the hybrids was calculated as follows: First, we calculated the genomic relationship for the maternal inbred lines and paternal inbred lines separately (VanRaden 2008). Assuming that the maternal and paternal inbred lines are unrelated, hybrid relationships were then calculated using the parental inbred relationship matrices as follows:

$$r_{ij} = 0.5*\left( {r_{{m_{i} ,m_{j} }} + r_{{p_{i} ,p_{j} }} } \right),$$

where $r_{ij}$ is the genomic relationship between hybrids $i$ and $j$, $r_{{m_{i} ,m_{j} }}$ is the genomic relationship between female parents of hybrid $i$ and $j$, $r_{{p_{i} ,p_{j} }}$ is the genomic relationship between male parents of hybrid $i$ and $j$.

A linear mixed model was used to estimate the genetic (co)variances for grain yield, grain moisture and plot mean NDVI, GNDVI, NDRE, SAVI, EVI and Ratio values at different time points.

A single-trait genomic best linear unbiased prediction (ST-GBLUP) model was fit to estimate the genetic and residual variances:

5 $${\mathbf{y}} = 1\mu + {\mathbf{Xb}} + {\mathbf{Za}} + {\mathbf{e}}$$

where ${\mathbf{y}}$ is the vector for the raw phenotype, *µ* is the overall mean, **b** is the vector of fixed effect of replicate, $a$ is the vector of random additive genetic effects of the hybrid to be estimated, **X** is the design matrix that allocates the fixed effect of replicate and **Z** is the design matrix that allocates additive genetic effects to observations, and ***e*** is the vector of residuals. It is assumed that $a\,\sim\,N\left( {0,\sigma_{a}^{2} {\mathbf{G}}} \right)$, where $\sigma_{a}^{2}$ is additive genetic variance and **G** is the genomic relationship matrix between the hybrids (VanRaden 2008), and that $e\,\sim\,N\left( {0,{\mathbf{I}}\varvec{\sigma}_{e}^{2} } \right)$, where ${\mathbf{I}}$ is the identity matrix and $\sigma_{e}^{2}$ is the variance of random residual effects. Narrow sense heritability was estimated for grain yield, grain moisture and the above-mentioned VIs as a ratio of estimated additive genetic variance to the sum of the additive genetic variance and residual variance estimated from the linear model.

Multi-trait GBLUP (MT-GBLUP) was fit to estimate the genetic and residual correlations between the above-mentioned end-of-season traits and mean values of the VIs at each time point. The general multi-trait model used was as follows:

6 $$\left[ {\begin{array}{*{20}c} {{\mathbf{y}}_{1} } \\ { {\mathbf{y}}_{2} } \\ \end{array} } \right] = \left[ {\begin{array}{*{20}c} {1\mu_{1} } \\ { 1\mu_{2} } \\ \end{array} } \right] + \left[ {\begin{array}{*{20}c} {{\mathbf{X}}_{1} 0 } \\ { 0 {\mathbf{X}}_{2} } \\ \end{array} } \right]\left[ {\begin{array}{*{20}c} {{\mathbf{b}}_{1} } \\ { {\mathbf{b}}_{2} } \\ \end{array} } \right] + \left[ {\begin{array}{*{20}c} {{\mathbf{Z}}_{1} 0 } \\ { 0 {\mathbf{Z}}_{2} } \\ \end{array} } \right]\left[ {\begin{array}{*{20}c} {{\mathbf{a}}_{1} } \\ { {\mathbf{a}}_{2} } \\ \end{array} } \right] + \left[ {\begin{array}{*{20}c} {{\mathbf{e}}_{1} } \\ { {\mathbf{e}}_{2} } \\ \end{array} } \right]$$

where **y**_**1**_ and **y**_**2**_ are the vectors of phenotypic values for traits 1 and 2; $\mu_{1}$ and $\mu_{2}$ are the overall means; $a_{1}$ and $a_{1}$ are the vectors of random additive genetic effects; ${\mathbf{X}}_{1}$ and ${\mathbf{X}}_{2}$ are the incidence matrices linking ${\mathbf{b}}_{1}$ to ${\mathbf{y}}_{1}$ and ${\mathbf{b}}_{2}$ to ${\mathbf{y}}_{2}$; ${\mathbf{Z}}_{1}$ and ${\mathbf{Z}}_{2}$ are the incidence matrices linking ${\mathbf{a}}_{1}$ to ${\mathbf{y}}_{1}$ and ${\mathbf{a}}_{2}$ to ${\mathbf{y}}_{2}$; ${\mathbf{e}}_{1}$ and ${\mathbf{e}}_{2}$ are vectors of random residual effects for trait 1 and 2, respectively. It was also assumed that $\left[ {{\mathbf{a}}_{1} {\mathbf{a}}_{2} } \right]\,\sim\,N\left( {0, {\varvec{\Sigma}} \otimes {\mathbf{G}}} \right)$ where $${\varvec{\Sigma}} = \left[ {\begin{array}{*{20}c} {\sigma_{{a_{1} }}^{2}\quad \sigma_{12} } \\ { \sigma_{21}\quad \sigma_{{a_{2} }}^{2} } \\ \end{array} } \right]$$ as unstructured genetic variance and covariance matrix of the traits, and **G** is the same as in Eq. 4, and $$\left[ {{\mathbf{e}}_{1} {\mathbf{e}}_{2} } \right]\,\sim\,N\left( {0,\left[ {\begin{array}{*{20}c} {\sigma_{{e_{1} }}^{2} \quad \sigma_{12} } \\ { \sigma_{21} \quad \sigma_{{e_{2} }}^{2} } \\ \end{array} } \right] \otimes {\mathbf{I}}} \right)$$ where **I** is the identity matrix.

For the bi-variate multi-trait model, one end-of-season trait was modeled jointly with a single VI. This model was fit for all combination of VI time points and end-of-season traits. In addition to the bi-variate model, multi-trait models including all time points for the VIs were fit using the 2016 and 2017 data. The model based on the 2017 data converged with all five time points in the multi-trait analysis; however, models fitting all time points for 2016 failed to converge.

### Random regression

Random regression models using linear splines were used to fit a model for all time points using mean NDVI values. The linear spline function is formed by connecting linear segments and the point at which the segments are connected are called knots of the spline. Since we have only four time points in 2015, the data from 2016 and 2017 were used to fit the random regression model.

The general random regression model for a single trait can be formulated as (Schaeffer 2004):

7 $$y_{ijn:t} = f_{j} + \mathop \sum \limits_{k = 1}^{\text{m}} a_{ki} z_{ijn:kt} + \mathop \sum \limits_{k = 1}^{\text{m}} pe_{kl} z_{ijn:kt} + e_{ijln:t} ,$$

where $y_{ijn:t}$ is the *n*th observation on *i*th hybrid at time *t* in the *j*th fixed factor; $f_{j}$ is a fixed effect of replicate by time that accounts for the mean growth curve; and in $\sum\nolimits_{k = 1}^{m} {a_{ki} } z_{ijn:kt} ,$ *m* is the number of the spline knot points, and $z_{ijn:kt}$ are the covariables related to time *t* at knot point *k,* $a_{ki}$ are the random regression coefficients for the *k*th knot for the *i*th hybrid, and in $\sum\nolimits_{k = 1}^{m} {{\text{pe}}_{kl} } z_{ijn:kt}$, ${\text{pe}}_{kl}$ is a random permanent environmental effect for the *k*th knot for plot *l* and $e_{ijln:t}$ is a random residual effect.

Growing degree days for each time points were calculated as:

$${\text{GDD}} = \frac{{T_{{{\rm max } }} + T_{\text{min }} }}{2} - T_{\text{base }} ,$$

where $T_{ \hbox{max} }$ is the maximum temperature, $T_{ \hbox{min} }$ is the minimum temperature and $T_{{{\text{base}} }} = 10 \,^\circ {\text{C}}$ is the base temperature. Any temperature below $T_{{{\text{base}} }}$ is set to $T_{\text{base}}$ before calculating the average. Likewise, the maximum temperature is set at 30 °C.

For a data point collected at time *t* and bounded by knots points $k$ and $\left( {k + 1} \right)$, the time covariables ($z_{{ijn:k{\text{t}} }}$ and $z_{{ijn:\left( {k + 1} \right)t }}$) in Eq. 7 were calculated as:

$$z_{ijn:kt } = \frac{{{\text{GDD}}_{k + 1} - {\text{GDD}}_{s} }}{{{\text{GDD}}_{k + 1} - {\text{GDD}}_{k} }} \,{\text{and}}\, z_{{ijn:\left( {k + 1} \right){\text{t}} }} = 1 - z_{{ijn:k{\text{t}} }}$$

where ${\text{GDD}}_{k}$, ${\text{GDD}}_{s}$ and ${\text{GDD}}_{k + 1}$ are the growing degree days at the time points for knot *k*, time point *s*. All knot points not flanking the data point at time *t* are set to zero.

The matrix notation of the random regression model is (Mrode and Thompson 2005):

8 $${\bf y} = {\mathbf{Xb}} + {\mathbf{Z}}_{1} {\mathbf{a}} + {\mathbf{Z}}_{2} {\mathbf{pe}} + e$$

where *y* is the vector of observation, ${\mathbf{X}}$ is the incidence matrix corresponding to fixed effect of replicate nested in time, ${\mathbf{b}}$ is the vector of fixed effects, ${\mathbf{Z}}_{1}$ is a matrix of regressors which are a function of the time point of the image capture, ${\mathbf{a}}$ is a vector of random genetic regression coefficients, ${\mathbf{Z}}_{2}$ is the matrix of regressors for each time point and ${\text{pe}}$ is the vector of random permanent environmental effect which is fit for every plot. The (co)variance matrix for the random genetic effect (*a*) is defined as:

$${\varvec{\Sigma}}_{{\mathbf{a}}} \otimes {\mathbf{G}} = \left[ {\begin{array}{*{20}c} {\sigma_{{{\text{a}}1}}^{2} } &\quad {\sigma_{{{\text{a}}1,{\text{a}}2}} } &\quad \ldots &\quad {\sigma_{{{\text{a}}m,{\text{a}}m}} } \\ {} & {\sigma_{{{\text{a}}2}}^{2} } & {} & {} \\ {} & {} &\quad \ddots & {} \\ {} & {} & {} &\quad {\sigma_{{{\text{a}}m}}^{2} } \\ \end{array} } \right] \otimes {\mathbf{G}}$$

where $\sigma_{{{\text{a}}1}}^{2}$ is the genetic variance at knot point $1$, $\sigma_{{{\text{a}}m}}^{2}$ is genetic variance at knot point $m$, (*m* = number of knot points), $\sigma_{{{\text{a}}1,{\text{a}}2}}$ is the genetic covariance at knot point $1$ and $2$ and **G** is the same as in Eq. 4. The (co)variance matrix for the random permanent environmental effect (${\text{pe}}$) is:

$${\varvec{\Sigma}}_{{{\mathbf{pe}}}} \otimes {\mathbf{I}} = \left[ {\begin{array}{*{20}c} {\sigma_{pe1}^{2} } & {\sigma_{pe1,pe2} } &\quad \ldots & {\sigma_{pem,pem} } \\ {} & \quad{\sigma_{pe2}^{2} } & {} & {} \\ {} & {} &\quad \ddots & {} \\ {} & {} & {} &\quad {\sigma_{pem}^{2} } \\ \end{array} } \right] \otimes {\mathbf{I}}$$

where diagonal elements in $${\varvec{\Sigma}}_{{{\mathbf{pe}}}}$$ are the variance components for permanent environment at each time point, the off-diagonals are the covariance of permanent environment between time points and **I** is the identity matrix. The diagonal (co)variance functions were defined for the random residual effect ($$e)$$ as follows:

$${\varvec{\Sigma}}_{{\mathbf{e}}} \otimes {\mathbf{I}} = \left[ {\begin{array}{*{20}c} {\sigma_{e1}^{2} } & {} & \ldots & {} \\ {} & {\sigma_{e2}^{2} } & {} & {} \\ {} & {} & \ddots & {} \\ {} & {} & {} & {\sigma_{et}^{2} } \\ \end{array} } \right] \otimes {\mathbf{I}},$$

where $\sigma_{e1}^{2}$ is the residual variance at time point $1$, $\sigma_{et}^{2}$ is the residual variance at time point $t$ and **I** is the identity matrix.

The genetic (co)variance between any combinations of time points for a given hybrid can be calculated as:

9 $$\sigma_{t }^{2} = \left[ {z_{1,t} z_{2,t} z_{3,t} } \right]*{\varvec{\Sigma}}_{{\mathbf{a}}} *\left[ {\begin{array}{*{20}c} {z_{1,t} } \\ {z_{2, t} } \\ {z_{3, t} } \\ \end{array} } \right] ;$$

and

10 $$\sigma_{t,s} = \left[ {z_{1,t} z_{2,t} z_{3,t} } \right]*{\varvec{\Sigma}}_{{\mathbf{a}}} *\left[ {\begin{array}{*{20}c} {z_{1,s} } \\ {z_{2,s} } \\ {z_{3,s} } \\ \end{array} } \right]$$

where ${\varvec{\Sigma}}_{{\mathbf{a}}}$ is the genetic (co)variance function for a linear spline with *m* = 3 knots, $\sigma_{T }^{2}$ is the genetic variance at time point *T*, $\sigma_{t,s}$ is the genetic covariance between time point *t* and *s*, and $z_{k,t}$ is the regression coefficients for time point $t$, at knot point $k$, and $z_{k, s}$ is the regression coefficients for time point $s$, at knot point *k*.

Among the five time points in 2016, the first two and the last two time points were used as knot points in the random regression model, whereas in 2017, the first, third and fifth (last) time points were used as knot points.

### Genomic prediction and cross-validation

ST- and MT-GBLUP was performed using fivefold cross-validation for grain yield and moisture. For ST-GBLUP, phenotypic data for grain yield and moisture were masked for 20% of the hybrids and thus used as a prediction set. In MT-GBLUP, the same fivefold cross-validation dataset that was used for ST-GBLUP was used. In this case, however, the phenotypic values of all the hybrids for one of the VIs at a given time point were used as a secondary trait. The prediction accuracy was calculated as the correlation between plot-level raw phenotypic value and the predicted genetic values. The cross-validation was nested within site/year because of differing experimental designs, planting and flight dates across site/years.

### Software

All data preparation, imputation of missing genotypes and construction of the genomic relationship matrix were performed in the R environment (R Development Core Team 2019), and the genetic analysis was performed using ASReml statistical software (Gilmour et al. 2015). Random regression analysis was performed using REMLF90 software (Misztal et al. 2002).

## Results

### Genetic variances and correlations

In order to investigate the amount of variation present in the VIs and understand their relationship with grain yield and moisture, genetic variance and correlation were evaluated using ST- and MT- models, respectively. Table 2 shows genetic and residual variances, together with the heritability estimates for grain yield and moisture for NYH2 and NYH3 sites in 2015 and at NYH2 in 2017. It should be noted that variance estimates obtained using GBLUP are not equivalent to those obtained using pedigree information, where genetic variance from the GBLUP model in particular has been shown to have unpredictable bias (de los Campos et al. 2015; Kumar et al. 2016); however, heritability estimates obtained from GBLUP were similar to those obtained when replacing G with and identity matrix (results not shown), indicating these estimates are a reasonable indicator of heritable variation.

**Table 2:** Within site/year additive ($\sigma_{a}^{2}$) and residual variance ($\sigma_{e}^{2}$) and heritability ($h^{2}$) for grain yield and grain moisture

Trait	2015 NYH2			2015 NYH3			2017 NYH2		
	$\sigma_{a}^{2}$	$\sigma_{e}^{2}$	$h^{2}$	$\sigma_{a}^{2}$	$\sigma_{e}^{2}$	$h^{2}$	$\sigma_{a}^{2}$	$\sigma_{e}^{2}$	$h^{2}$
Grain yield^a^	509.30	862.37	0.40	607.70	792.10	0.43	308.84	684.40	0.31
Grain moisture^a^	3.20	0.90	0.80	7.655	1.621	0.82	4.89	0.62	0.89

^a^Grain yield is the amount of grain harvested per plot and it is measured as bushel per acreage. Grain moisture is measured as the amount of moisture content in grains harvested per plot and it is measured as percent moisture

Table 3 shows the heritability estimates for mean NDVI, GNDVI, NDRE, SAVI, EVI and Ratio at different growth stages in 2015 at NYH2 and NYH3, and in 2016 and 2017 at NYH2. Results indicate that there is considerable amount of heritable variation in different VIs.

**Table 3:** Within site/year heritability estimates for NDVI, GNDVI, NDRE, SAVI, EVI, and Ratio at different time points and their standard errors as a subscript

Growth stage	GDD	NDVI	GNDVI	NDRE	SAVI	EVI	Ratio
*2015 NYH2*							
R1–R2	686	0.16_0.07_	0.10_0.06_	0.16_0.07_	0.20_0.07_	0.20_0.07_	0.43_0.07_
R1–R2	882	0.20_0.08_	0.12_0.07_	0.13_0.07_	0.32_0.08_	0.30_0.07_	0.36_0.06_
R3–R4	1037	0.40_0.08_	0.25_0.08_	0.23_0.08_	0.42_0.07_	0.41_0.07_	0.50_0.06_
R5–R6	1272	0.34_0.07_	0.05_0.03_	0.04_0.04_	0.12_0.05_	0.13_0.08_	0.10_0.04_
*2015 NYH3*							
VT	554	0.10_0.06_	0.10_0.06_	0.10_0.05_	0.10_0.04_	0.10_0.04_	0.00_0.00_
R1–R2	759	0.36_0.07_	0.32_0.07_	0.33_0.07_	0.31_0.07_	0.32_0.07_	0.33_0.07_
R1–R2	916	0.50_0.06_	0.31_0.07_	0.27_0.07_	0.21_0.07_	0.23_0.07_	0.35_0.07_
R3–R4	1157	0.50_0.06_	0.28_0.07_	0.33_0.07_	0.62_0.05_	0.62_0.05_	0.38_0.06_
*2016 NYH2*							
VT	524	0.32_0.07_	0.32_0.07_	0.30_0.07_	0.21_0.06_	0.22_0.07_	0.26_0.07_
R1–R2	816	0.32_0.08_	0.37_0.08_	0.36_0.07_	0.23_0.07_	0.30_0.08_	0.33_0.07_
R3–R4	1129	0.50_0.07_	0.41_0.07_	0.46_0.07_	0.54_0.06_	0.53_0.06_	0.48_0.07_
R3–R4	1173	0.60_0.06_	0.58_0.06_	0.58_0.06_	0.60_0.06_	0.61_0.05_	0.62_0.05_
R5–R6	1501	0.77_0.04_	0.69_0.05_	0.80_0.03_	0.78_0.03_	0.78_0.03_	0.80_0.03_
*2017 NYH2*							
R1–R2	814	0.25_0.07_	0.10_0.04_	0.10_0.03_	0.33_0.07_	0.30_0.07_	0.25_0.08_
R3–R4	974	0.45_0.07_	0.21_0.06_	0.18_0.06_	0.59_0.06_	0.59_0.06_	0.50_0.07_
R3–R4	1102	0.58_0.07_	0.26_0.07_	0.25_0.07_	0.67_0.05_	0.66_0.05_	0.56_0.06_
R5–R6	1135	0.62_0.05_	0.31_0.07_	0.33_0.07_	0.73_0.04_	0.73_0.04_	0.50_0.07_
R5–R6	1167	0.82_0.03_	0.32_0.07_	0.44_0.07_	0.87_0.02_	0.87_0.02_	0.84_0.03_

In 2015 at location NYH2, lower heritability estimates were observed at the late reproductive stage for all the VIs except for NDVI, where lower heritability was observed at the early reproductive stage (R1–R2). For all the VIs, the maximum heritability estimate was obtained at the mid-reproductive stage (R3–R4) and the highest heritability was observed for Ratio. At NYH3, the heritability estimates for NDVI were found to have an increasing pattern through the growth stages. The same is true for NDRE and Ratio. For GNDVI, SAVI and EVI, no clear pattern was observed.

For 2016 and 2017, the heritability estimates for all VIs show an increasing pattern through growth stages, with the highest estimate observed at the late reproductive stage (R3–R4).

In order to investigate the relationship between different VIs and important end-of-season traits, genetic and residual correlations were estimated for the two end-of-season traits (grain yield and moisture) and all the VIs. However, correlation results are presented only for NDVI and NDRE. This is because NDVI is the most commonly used VI to measure important crop phenotypes and it was found to have consistently high heritability across site/year and genetic correlation with the end-of-season traits in this study, and NDRE tends to be less prone to saturation at a maximum value of 1 (Cammarano et al. 2014). Genetic correlation between the rest of the VIs (EVI, GNDVI, SAVI and Ratio) and the end-of-season traits at each site/year is presented in “Appendix 1.”

**Fig. 1:**
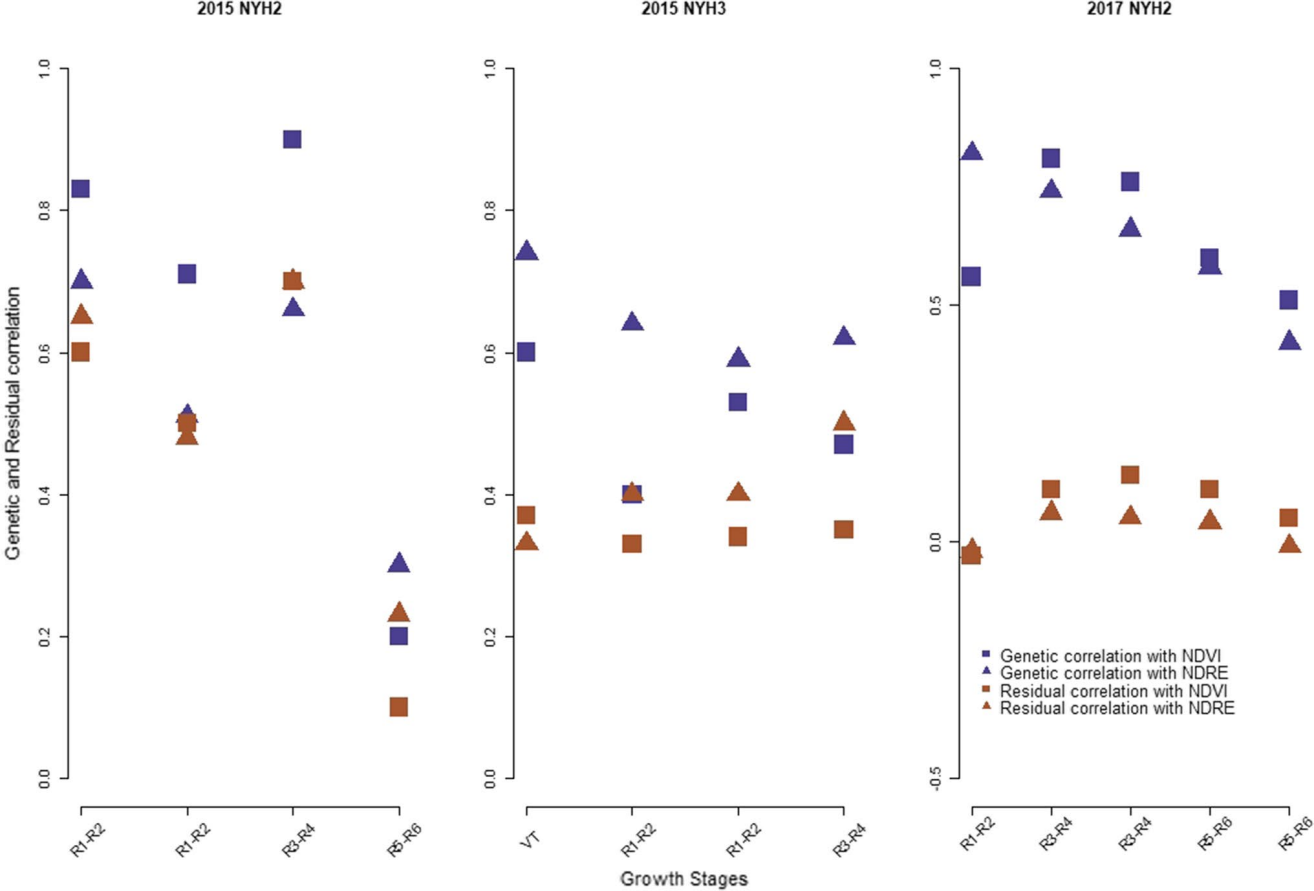
Genetic and residual correlation of grain yield with NDVI and NDRE at different time points in 2015 at NYH2 and NYH3 and in 2017 at NYH2

Figure 1 shows the genetic and residual correlation of grain yield with NDVI and NDRE at different growth stages in 2015 at NYH2 and NYH3, and in 2017 at NYH2. In 2015 at NYH2 and NYH3, both genetic and residual correlations of grain yield with NDVI and NDRE were positive and range from moderate to very strong at different growth stages. In 2017, however, stronger genetic correlations of grain yield with NDVI and NDRE were observed during the early reproductive stage (R1–R2) and show a decreasing pattern in the later reproductive stages (R3–R6). Weaker residual correlations were observed in 2017 when compared to the residual correlations in 2015.

**Fig. 2:**
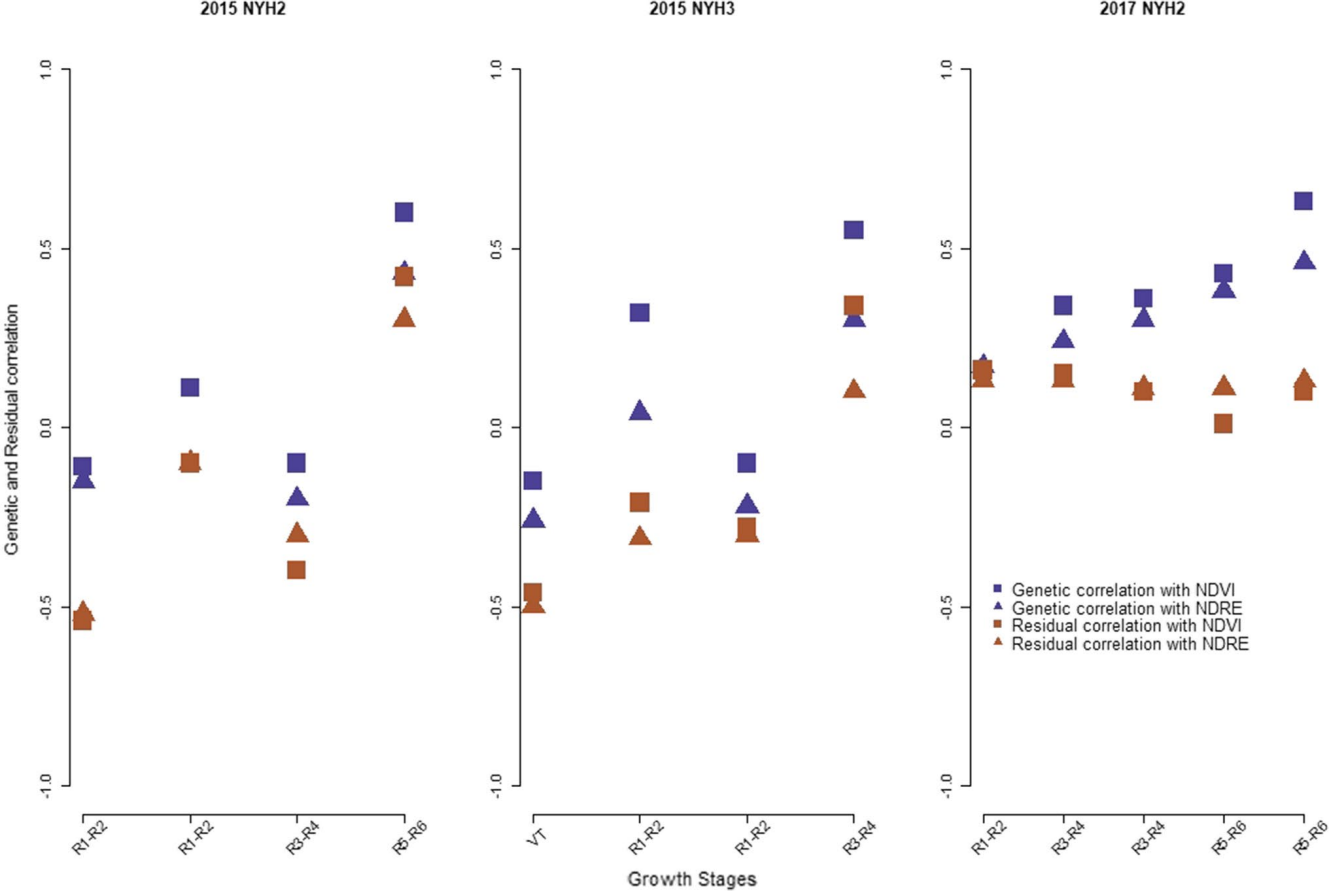
Genetic and residual correlation of grain moisture with NDVI and NDRE at different time points in 2015 at NYH2 and NYH3, and in 2017 at NYH2

Figure 2 shows the genetic and residual correlation of grain moisture with NDVI and NDRE at different growth stages in 2015 at NYH2 and NYH3 and in 2017 at NYH2. In 2015 at NYH2 and NYH3, genetic and residual correlations with both of the VIs were negative and weak during the late vegetative stages (VT) and early reproductive stage (R1–R2). At the later reproductive stages, however, positive and moderately strong correlations were observed. The same is true for the residual correlation at both locations, where negative correlations were observed during the late vegetative stage/early reproductive stage and positive at later growth stages. In 2017 at NYH2, genetic correlation with NDVI and NDRE was high and positive throughout different reproductive stages. The residual correlations, on the other hand, were very weak or close to zero.

### Accuracy of genomic prediction

**Fig. 3:**
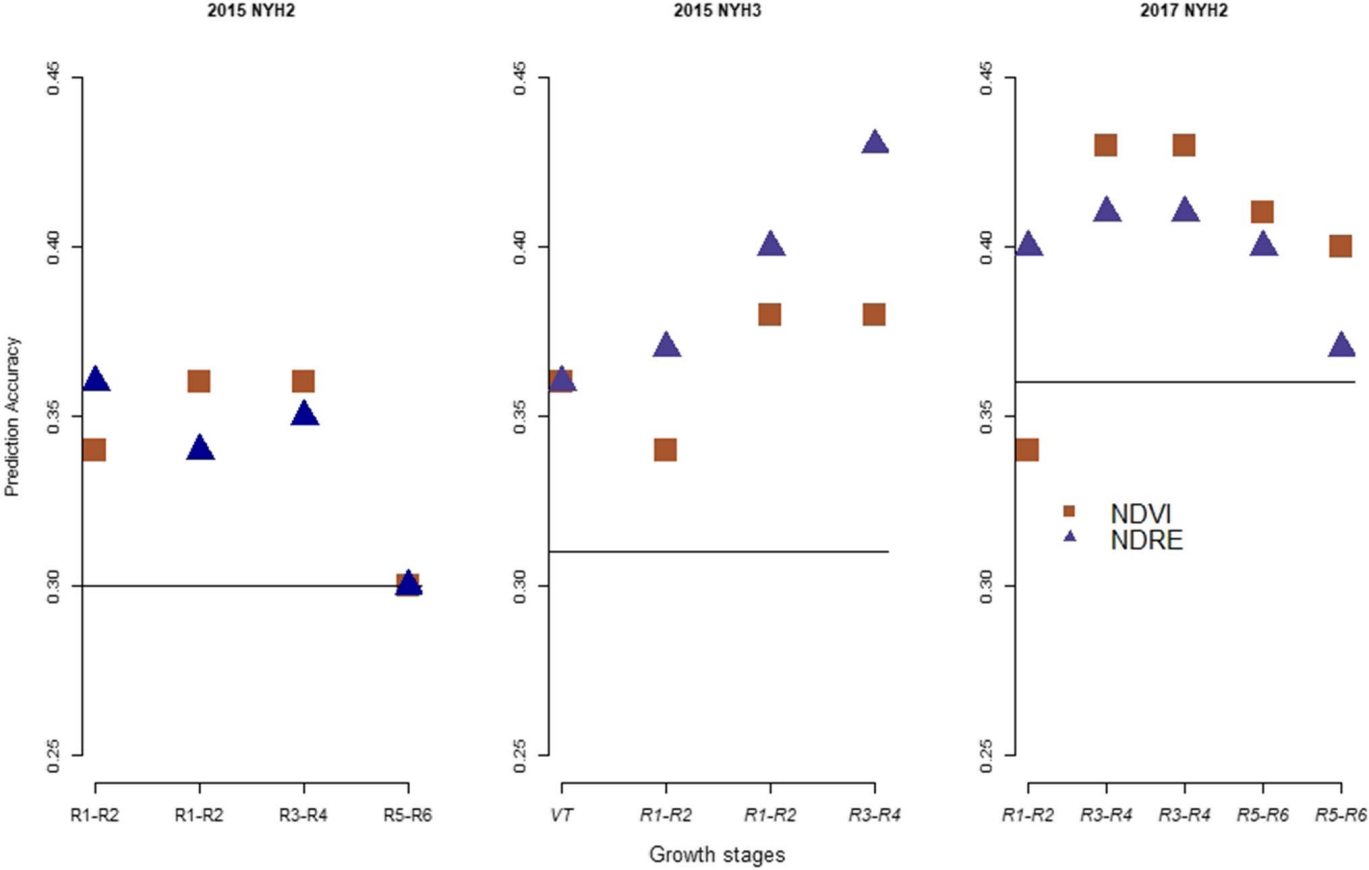
Prediction accuracy from ST-GBLUP (horizontal line) and MT-GBLUP model for grain yield when NDVI or NDRE was used as a secondary trait in 2015 at NYH2, NYH3 and in 2017 at NYH2. These results are averages from fivefold cross-validation

**Fig. 4:**
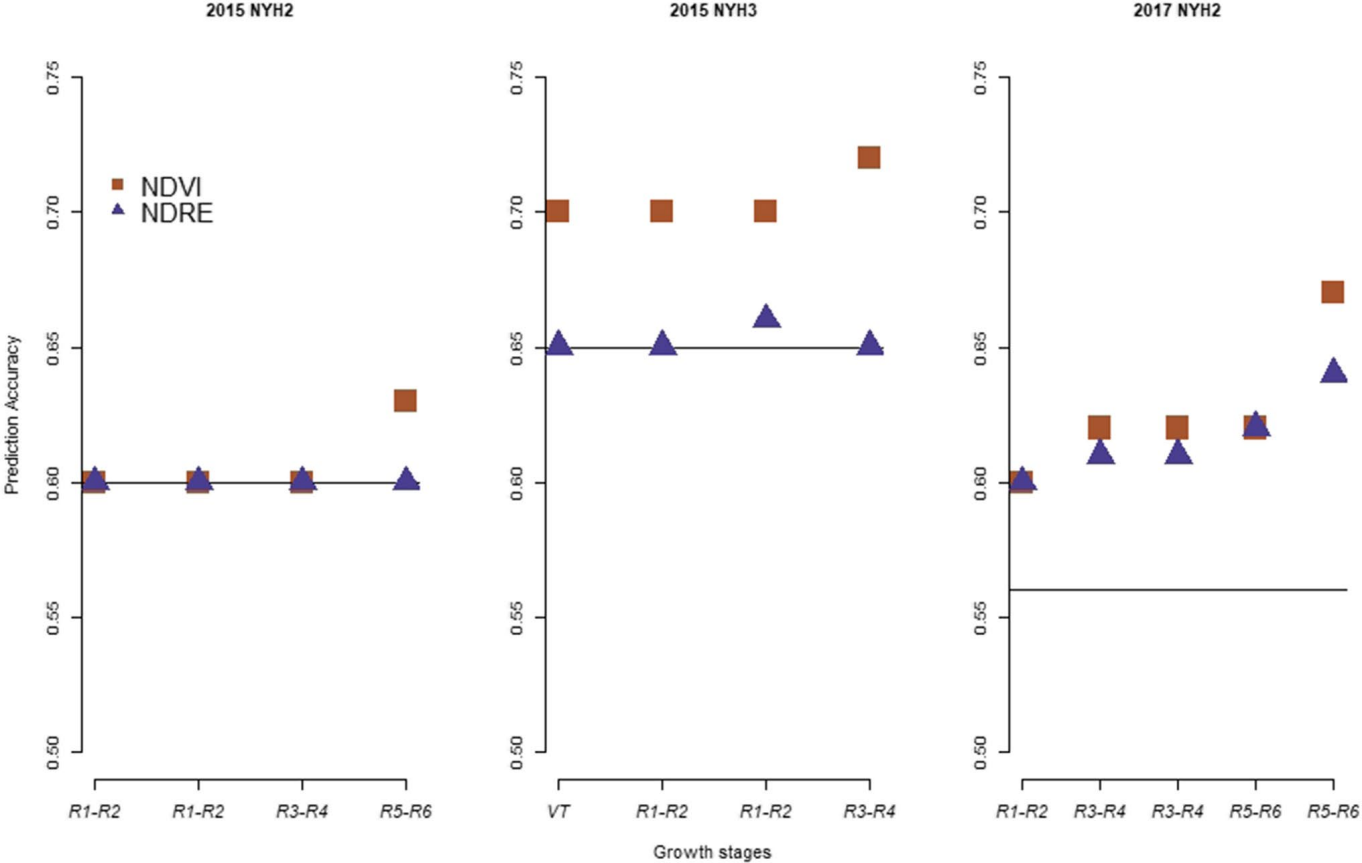
Prediction accuracy from ST-GBLUP (horizontal line) and MT-GBLUP model for grain moisture when NDVI or NDRE was used as a secondary trait at different growth stages in 2015 at NYH2, NYH3 and in 2017 at NYH2. These results are averages from fivefold cross-validation

In order to investigate the advantage of using VIs as a secondary trait, multi-trait genomic prediction for grain yield and moisture was performed. Prediction accuracies from the multi-trait model for grain yield and moisture in reference to the prediction accuracy from the single-trait model are presented in Figs. 3 and 4. In this figure, prediction accuracy from ST-GBLUP model is presented as a horizontal line, and results are averages from fivefold cross-validation. Prediction accuracy from the multi-trait models is presented only when NDVI or NDRE was used as a secondary trait.

Figure 3 shows prediction accuracies for grain yield from the MT-GBLUP model, in 2015 at NYH2 and NYH3 and in 2017 at NYH2. In 2015 at NYH2, a considerable amount of gain (17–20%) in prediction accuracy from multi-trait model was observed at the early (R1–R2) and mid (R3–R4)- reproductive stages. This is true when either of the two VIs was used as a secondary trait. At later reproductive stages, however, there was no gain in prediction accuracy from the multi-trait models. In 2015 at NYH3, substantial increase (15–40%) in prediction accuracies from multi-trait model was observed at all growth stages and when either of the two VIs was used as a secondary trait. In 2017 at NYH2, considerable increases in prediction accuracies (70–100%) were obtained from the multi-trait model throughout different growth stages.

Figure 4 shows prediction accuracies for grain moisture in 2015 at NYH2 and NYH3 and in 2017 at NYH2. In 2015 at NYH2, there was no gain in prediction accuracies from multi-trait models during the early (R1–R2) and mid (R3–R4)- reproductive stages. A smaller increase in prediction accuracy was observed at late (R5–R6) stages and only when NDVI was used as a secondary trait. In 2015 at NYH3, significant increases (7–10%) in prediction accuracy were obtained from the multi-trait model. This is, however, true only when NDVI was used as a secondary trait, with zero to small gains in prediction accuracy observed when NDRE was used as a secondary trait. In 2017 at NYH2, increases in prediction accuracy from multi-trait model were significant (7–20%) when either of the two VIs was used as a secondary trait.

### Random regression

**Fig. 5:**
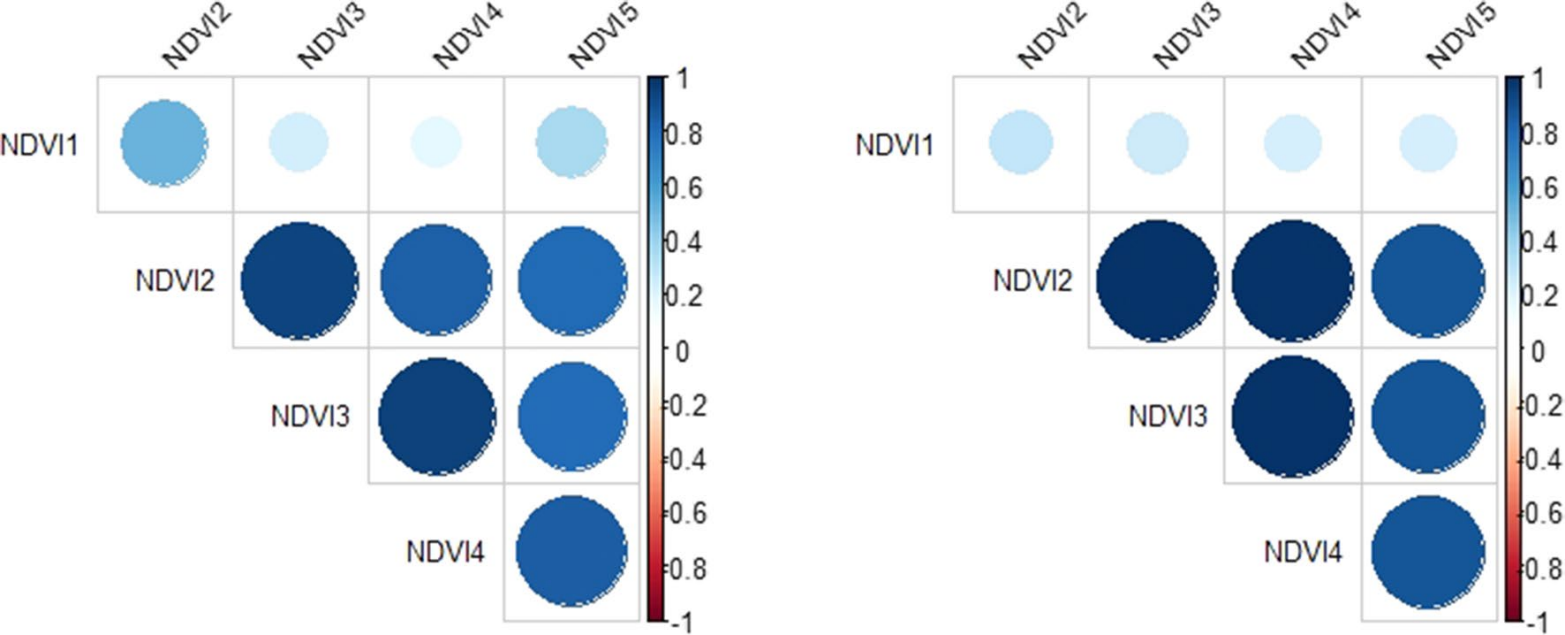
Estimated genetic correlation from multi-trait mixed model (left) and constructed genetic correlations (right) between NDVI values at different time points in 2017. The size of the circles indicate the degree of correlation and the colors indicate the direction of the correlation

**Fig. 6:**
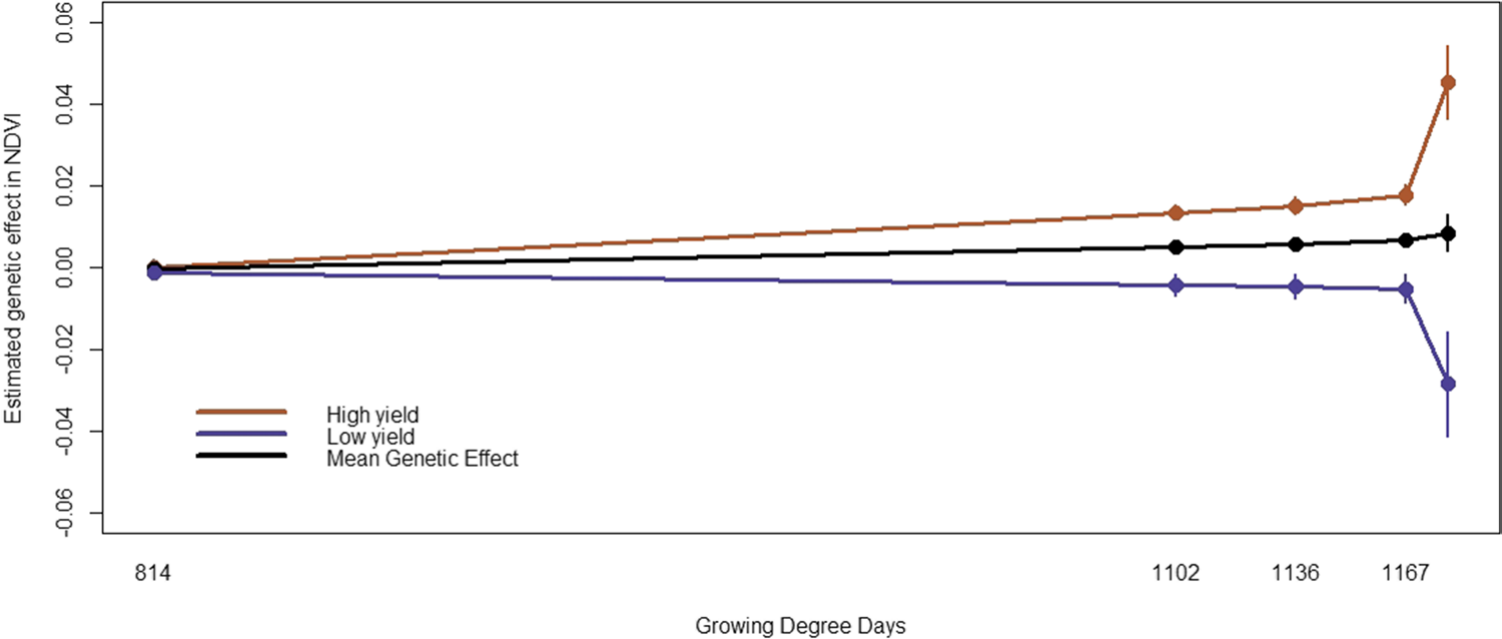
Mean estimated genetic effect for NDVI curve for the high (201–270 bu/acre) and low (70–120 bu/acre) yielding hybrids and for population mean across the growing degree days in 2017

In 2016, the first two and the last two time points were fit as knot points, and in 2017 the first, fourth and last time points were fit as knot points in the random regression model. The random regression models fit permanent environmental effects to account persistent environmental effect associated with a given plot. The permanent environmental effects account for the correlated residuals observed when fitting the multi-trait models. The genetic effect estimates from the random regression model at these time points ($b_{ki}$) were correlated with their counter estimates from the multi-trait mixed model ($a_{i}$). For 2016, the correlation at the four knot points was 0.99, 0.99, 0.99 and 0.99. Given the multi-trait model did not converge for 2016 using all data, these correlations were obtained by comparing to a multi-trait model including only the times used a knots. In 2017, since the multi-trait model with all the time points was able to converge, the correlations at all the time points were estimated. The correlations at all five time points were 0.92, 0.96, 0.97, 0.98 and 0.99. Moreover, (co)variance structure for all the time points was constructed using Eqs. 9 and 10 and the covariance structure between all the time points estimated from the multi-trait mixed model was compared to the covariance structure that was constructed from the covariance function and is found in Fig. 5. As it can be seen from the correlation structures (Fig. 5), the constructed correlation structure (right) from the spline covariance function approximates very well to the full correlation structure (left) that was estimated from the multi-trait mixed model. Figure 6 shows mean NDVI curves of random regression estimates for high- and low-yielding lines in 2017. As it can be seen from the figure, the high-yielding hybrids tend to have high NDVI values and the low yielding hybrids tend to have lower NDVI value throughout the growing degree days.

## Discussion

The main objective of this study was to investigate the amount of variation present in several VIs derived from MSI and the relationship between growth curves, as measured by VIs, and end-of-season traits. Results indicate that variations in growth curves are heritable, ranging from low to high heritabilities, and genetically correlated with important end-of-season traits. Similar results were observed by Sun et al. (2017), where moderate to higher heritability estimates were observed for NDVI and NDRE across different locations in wheat.

In 2015 at NYH3, and in 2016 and 2017 at NYH2, the heritability estimates for all the VIs were lower at the early reproductive stage, as was the total phenotypic variability (0.005–0.001) compared to the total phenotypic variability at the mid- and late reproductive stages (0.01–0.002), indicating the MSI was not able to detect variability between plots at this stage. The heritability estimates at the mid-reproductive stage in 2015 at NYH3 and at late reproductive stage in 2016 and 2017 at NYH2 were quite high, which could be due to the differentiation of the early/mid- and late maturing hybrids. These results suggest that mid- and late reproductive stages are important time points for the collection of MSI and differentiation of growth curves between hybrids.

Strong genetic correlations (− 0.50 to 0.90) were found between the two VIs and the end-of-season traits, especially with grain yield, throughout different growth stages and sites. These high correlations throughout the growing season suggest VIs derived from UAV platforms have the potential to be used as an indicator trait in phenotypic selection and also to improve genomic prediction accuracy.

As observed in Figs. 3 and 4, the amount of prediction accuracy from the multi-trait model for grain yield and moisture was considerable. This was the case at most of the time points in 2015 and 2017, and when either NDVI or NDRE was used as a secondary trait. Similar results were observed by (Rutkoski et al. 2016), where gain in prediction accuracy from multi-trait models was observed when either canopy temperature, NDVI or GNDVI was used as a secondary trait.

**Fig. 7:**
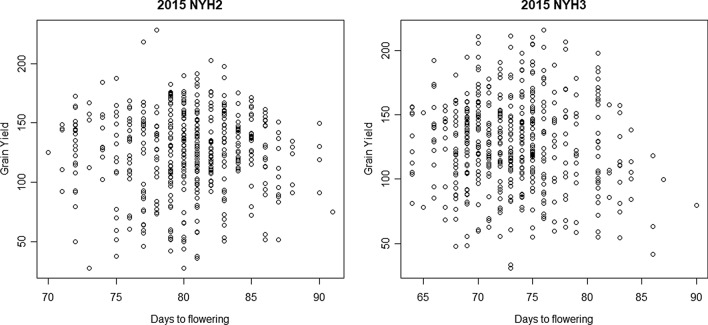
Grain yield for different days to flowering in 2015 at NYH2 and NYH3

**Fig. 8:**
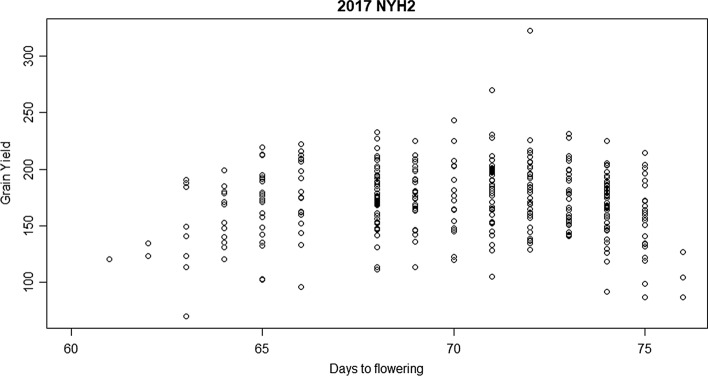
Grain yield at different days to flowering in 2017 at NYH2

It has been reported in wheat that when correcting grain yield for days to heading, the amount of prediction accuracy that can be obtained using VIs as a secondary trait reduces (Rutkoski et al. 2016; Krause et al. 2019). This suggests that some of the predictive power of vegetative indices comes through indirect correlations with maturity when grain yield and maturity are genetically correlated. In this study, grain yield was corrected for days to flowering for all sites and years included in the study and multi-trait genetic analysis was conducted between corrected grain yield and the two VIs NDVI and NDRE. For data collected in 2015, the correlation between VIs and grain yield shows no reduction after correcting for days to flowering. However, for data collected in 2017, significant change in genetic correlations between grain yield and the two VIs was observed, suggesting that genetic correlations could be sensitive to correction on days to flowering. These are expected since no relationship was observed between days to flowering and grain yield for the data collected in 2015 (Fig. 7). For the 2017 data, however, there was an observed relationship between days to flowering and yield (Fig. 8). Nevertheless, more data from diverse germplasm evaluated across years and locations are necessary in order to gain a better understanding of the relationship among grain yield, maturity and VIs across a range of genetic and environmental factors.

Multi-trait genomic prediction takes advantage of the correlation between the traits that are under consideration. Moreover, it was reported that low heritable traits tended to benefit more from multi-trait genomic prediction more than relatively highly heritable traits, in terms of accuracy of estimated breeding values (Mrode and Thompson 2005). In this study, this was hardly the case for grain moisture, where heritability for this trait was higher than the heritability of the three VIs at most of the growth stages in 2015 and 2017. This could be the reason for the limited amount of increase in prediction accuracy that was obtained from multi-trait genomic prediction at most of the time points, where no to very small gains in accuracy were observed for grain moisture. For grain yield, with a lower heritability, the increases in accuracy from the multi-trait model were substantial at almost all time points. These results suggest that predictions using VIs could be useful in environments where harvesting is difficult/impossible due to poor weather conditions, and the VIs could be used to decrease the cost of early stage multi-environmental testing by eliminating the need to measure end-of-season traits at all locations. The VIs also provide insight into heritable difference in development curves which could be related to genotype-by-environmental interactions. Information on growth curves may provide additional information for selecting lines that are best adapted to a given target population of environments or demonstrate increased stability in performance across varied conditions. However, given the limitations of multi-trait models to model a large number of time points, random regression models are a better option for modeling growth curves.

**Fig. 9:**
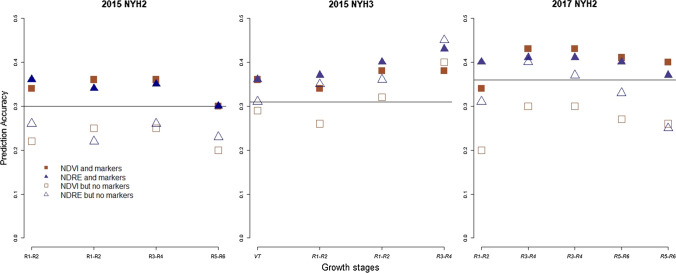
Prediction accuracy from ST-GBLUP (horizontal line) and MT-GBLUP model for grain yield when NDVI or NDRE was used as a secondary trait at different growth stages in 2015 at NYH2, NYH3 and in 2017 at NYH2. Solid plots indicate when marker information was used and open plots when no marker information was used. These results are averages from fivefold cross-validation

**Fig. 10:**
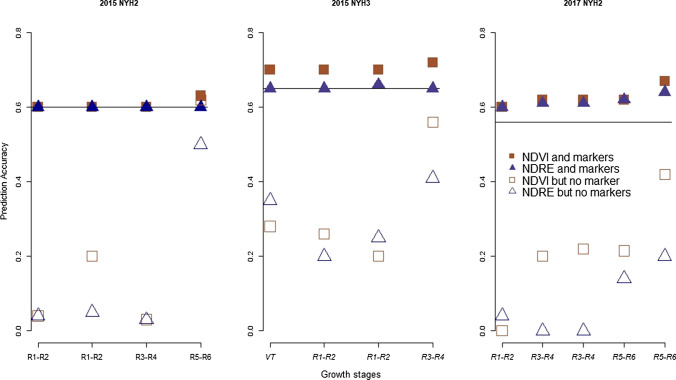
Prediction accuracy from ST-GBLUP (horizontal line) and MT-GBLUP model for grain moisture when NDVI or NDRE was used as a secondary trait at different growth stages in 2015 at NYH2, NYH3 and in 2017 at NYH2. Solid plots indicate when marker information was used and open plots when no marker information was used. These results are averages from fivefold cross-validation

Multi-trait predictions where NDVI and NDRE were fit as a secondary trait but without the use of marker information were fit for grain yield and moisture. For grain yield, the amount of gain obtained from a multi-trait model without the marker information was significant only in 2015 at NYH3 (Fig. 9). For grain moisture, the prediction accuracy from the multi-trait model without the marker information was lower than the prediction accuracy obtained from the single-trait genomic prediction at almost all stages of growth and sites/years (Fig. 10). These results suggest that the use of VIs alone may not be adequate to achieve the best predictions of grain yield and moisture.

The convergence issues encountered in the analysis of all VIs collected in 2016 using multi-trait mixed model highlight the limitations of this approach. As the number of time points and frequency of flights increase, convergence issues are likely to occur due to high correlation between the records at multiple time points and the large number of parameters that need to be estimated. One of the benefits of random regression models is the ability to partition the sources of variation in the trait of interest, such as genetic and permanent environmental variation, and model the change in these sources of variations as a function of time, thus reducing the number of parameters to be estimated. As a result, random regression models have been used to model longitudinal data for a variety of time dependent traits (Schaeffer 2004). This of course assumes that changes in (co)variance are some function of time.

In this study, a linear spline function was used to model changes in (co)variance for NDVI curves through time. The performance of the spline covariance function was evaluated by comparing (co)variance and genetic effect estimates to a multi-trait model including all time points in 2017. In general, the strong correlations observed between the random regression estimates and the mixed model estimates indicate similar performance when the number of time points included is relatively small. This suggests that random regression models using spline covariance functions can adequately model changes in (co)variance over time. Furthermore, the correspondence between patterns observed in the NDVI growth curves and yield measurements in 2017 (Fig. 6) indicates that trend in growth curves is related to end-of-season yield measurements.

It was reported that the optimal position of the spline knots varies between the genetic effect and permanent environmental effects, as well as between different traits (Jamrozik et al. 2010). Given the limited number of time points in this study, there were few options for optimizing the placement of knots; however, as the number of time points increases, knot placement will become increasingly important for optimal model fits.

The linear spline covariance function was selected for this study because of its simplicity and the sparse nature of the MSI data. As new datasets are generated with MSI collected frequently throughout the growing season, it will be possible to implement covariance functions using orthogonal polynomials to compare model fit for multiple covariance functions. Despite the sparsity of MSI images in this study, the multi-trait approach failed to converge using all MSI phenotypes, whereas the random regression models converged using all available data. As the number of MSI phenotypes increases, random regression models should provide a robust approach to leverage MSI collected at all time points to model growth curves, and given the large genetic correlations between images taken at different growth stages, this approach should provide more accurate estimates of genetic parameters of growth curves.

## Conclusion

Considerable heritable variation exists in all the VIs across sites and years. Furthermore, strong genetic correlations were found between grain yield, grain moisture and the VIs, resulting in moderate to large increases in prediction accuracies for grain yield and moisture when selected VIs were used as a secondary trait. The heritable variation in growth curves, as measured using MSI, and strong genetic correlations with economically important traits indicate that routine collection of MSIs throughout the growing season could be valuable for selection in breeding programs. While multi-trait models provide a straightforward approach to model end-of-season traits and VIs at multiple time points, convergence issues were encountered as the number of time points increase. In contrast, random regression models converged using all time points and appear to be a promising approach for modeling genetic components of growth curves when MSI is captured frequently throughout the growing season. Further research is needed to determine optimal covariance functions and best approaches for continually modeling changes in genetic covariance between VIs and end-of-season traits throughout the growing season.

## Appendix 1

See Tables 4, 5, 6 and Figs. 7, 8, 9, 10.

**Table 4:** Genetic correlation between EVI, GNDVI, SAVI and Ratio and end-of-season traits, grain yield and moisture at different growth stages in 2015 at NYH2

	R1–R2	R1–R2	R3–R4	R5–R6
*EVI*				
Grain yield	0.60	0.65	0.42	0.61
Grain moisture	− 0.03	0.25	0.37	0.91
*GNDVI*				
Grain yield	0.83	0.62	0.36	− 0.20
Grain moisture	− 0.14	− 0.34	− 0.23	0.20
*SAVI*				
Grain yield	0.53	0.40	0.61	0.72
Grain moisture	− 0.10	0.40	0.30	0.93
*Ratio*				
Grain yield	0.13	0.10	0.51	0.40
Grain moisture	0.10	− 0.01	− 0.10	0.34

**Table 5:** Genetic correlation between EVI, GNDVI, SAVI and Ratio and end-of-season traits, grain yield and moisture at different growth stages in 2015 at NYH3

	VT	R1–R2	R1–R2	R3–R4
*EVI*				
Grain yield	0.32	0.41	0.51	0.45
Grain moisture	− 0.30	0.41	0.43	0.62
*GNDVI*				
Grain yield	0.76	0.63	0.55	0.66
Grain moisture	− 0.21	− 0.02	− 0.32	0.12
*SAVI*				
Grain yield	0.26	0.30	0.43	0.41
Grain moisture	− 0.30	0.35	0.50	0.63
*Ratio*				
Grain yield	0.98	− 0.001	0.41	0.58
Grain moisture	0.97	0.20	− 0.20	0.44

**Table 6:** Genetic correlation between EVI, GNDVI, SAVI and ratio and end-of-season traits, grain yield and moisture at different growth stages in 2017 at NYH2

	R1–R2	R3–R4	R3–R4	R5–R6	R5–R6
*EVI*					
Grain yield	0.20	0.63	0.70	0.60	0.53
Grain moisture	0.21	0.50	0.52	0.57	0.67
*GNDVI*					
Grain yield	0.76	0.80	0.76	0.70	0.55
Grain moisture	0.20	0.33	0.40	0.43	0.50
*SAVI*					
Grain yield	0.12	0.55	0.64	0.56	0.51
Grain moisture	0.20	0.50	0.50	0.57	0.67
*Ratio*					
Grain yield	0.46	0.87	0.84	0.68	0.50
Grain moisture	0.17	0.36	0.40	0.40	0.65
